# *Cryptosporidium parvum* and bovine coronavirus in naturally and experimentally exposed calves: clinical outcome and pathogen shedding

**DOI:** 10.1186/s13567-026-01725-x

**Published:** 2026-04-03

**Authors:** Mathilde S. Varegg, Maria Stokstad, Paul M. Bartley, Frank Katzer, Lucy J. Robertson, Alejandro Jiménez-Meléndez

**Affiliations:** 1 Department of Paraclinical Sciences, Faculty of Veterinary Medicine, Norwegian University of Life Sciences, Elizabeth Stephansens vei 15, 1433 Aas, Norway; 2 Department of Production Animal Clinical Sciences, Faculty of Veterinary Medicine, Norwegian University of Life Sciences, Elizabeth Stephansens vei 15, 1433 Aas, Norway; 3 Moredun Research Institute, Pentlands Science Park, Bush Loan, Penicuik, Midlothian, EH26 0PZ Scotland, UK

**Keywords:** Neonatal calf diarrhea, enteropathogens, neonatal calves, *Cryptosporidium parvum*, bovine coronavirus, mixed infection, co-infection, pathogen shedding, clinical score, in vivo infection study

## Abstract

A comparative infection study was performed to investigate the clinical outcome and pathogen shedding patterns in calves experimentally infected with *Cryptosporidium parvum* and/or bovine coronavirus (BCoV). A total of 15 calves were enrolled and assigned into three infection groups (each *n* = 5) within 12 h of birth. Group 1 (G1) was experimentally infected with *C. parvum*, group 2 (G2) was experimentally infected with *C. parvum* and BCoV, and group 3 (G3) was experimentally infected with BCoV. Clinical outcomes were registered, and feces and nasal swabs were collected daily for 28 days. Prior to experimental infection, all calves were found negative for BCoV RNA in fecal and upper respiratory samples, but 12/15 calves had detectable *C. parvum* DNA in feces, meaning all groups were already naturally infected with *C. parvum*. Calves double infected with *C. parvum* (G1 and G2; experimentally and naturally infected) showed more clinical signs over a longer period of time, compared with calves experimentally infected with BCoV and also naturally infected with *C. parvum* (G3). The shedding pattern of *Cryptosporidium* DNA in feces was similar in all groups. The peak shedding period of *C. parvum* correlated with the days when calves displayed the most clinical signs. However, a significant increase in fecal BCoV RNA occurred after the period where calves displayed the most clinical signs. In this study, a double infection of neonatal calves with *C. parvum* (G1 and G2) resulted in a more severe clinical outcome than a mixed infection with *C. parvum* and BCoV.

**Supplementary Information:**

The online version contains supplementary material available at 10.1186/s13567-026-01725-x.

## Introduction

Neonatal calf diarrhea (NCD) is a multifactorial disease impacting the welfare of young calves globally. The neonatal period in calves is defined as the first month of life [1], and diarrhea during this period is a leading cause of morbidity and mortality in calves [2, 3]. The clinical presentation of NCD can range from mild to severe disease with general depression, dehydration, and death [4] and can severely impair animal welfare and negatively influence the long-term production of the animal later in life [5]. Several pathogens are associated with NCD, including *Cryptosporidium parvum*, bovine rotavirus, *Escherichia coli* (K99), and bovine coronavirus [6]. Despite decades of research and efforts to prevent and treat NCD, it remains a significant, intransigent obstacle to an ethical and sustainable cattle industry.

*Cryptosporidium parvum* is a protozoan parasite causing gastrointestinal disease in many species, including humans, with calves being the main reservoir [7]. In cattle, the parasite occurs frequently in calves, where it invades and destroys the intestinal epithelium, leading to malabsorptive NCD [8]. Bovine coronavirus (BCoV) of the genus *Betacoronaviridae* is a pneumoenteric virus causing both respiratory disease and enteric disease. Bovine coronavirus infection most commonly presents as three different syndromes in the cattle industry; NCD, respiratory disease in feedlot calves, and winter dysentery in adult cattle. As a causative agent of NCD, BCoV produces a malabsorptive diarrhea, which is often severe, and sometimes bloody [9].

Mixed infections with commonly occurring enteropathogens causing NCD are often observed; a global meta-analysis of calves with NCD caused by co-infections found prevalences involving *Cryptosporidium* spp. and BCoV of up to 6.7% in the USA and 4.3% in the UK [10]. Interactions between enteropathogens causing NCD have been observed in another global meta-analysis of calves with NCD of mixed origin with emphasis on bovine rotavirus, where they found that BCoV was more likely to be found in calves with diarrhea if bovine rotavirus was also present [11]. A synergistic interaction between BCoV and rotavirus was also observed in a study investigating dairy calf health in a veal-rearing facility in Canada, where co-infections of BCoV and rotavirus were associated with a higher proportion of days with severe diarrhea [12]. However, the likelihood of detecting BCoV, rotavirus, and *E. coli* in calves with diarrhea was reduced if *Cryptosporidium* was present [10]. These observations suggest that mixed infections affect the clinical outcome in calves differently, on the basis of which enteropathogens are present. Most reports on interactions in mixed infections causing NCD focus on *C. parvum* and/or rotavirus [10, 11, 13, 14], and as far as we are aware, there is limited information about how mixed infections with *C. parvum* and bovine coronavirus affect the health of neonatal calves. In vitro studies have suggested that *C. parvum* could be a means of transport of BCoV into epithelial cells, but whether this effect occurs in vivo or how co-infection with *C. parvum* and BCoV might affect the clinical status of the animals remains unknown [15].

Further investigation of mixed infections would help to understand the clinical impact of different combinations of enteropathogens on neonatal calves. Studying mixed infections could increase basic knowledge of NCD pathogenesis and help farmers and veterinarians choose the most effective preventive measures in problem herds. For these investigations, a controlled environment and close surveillance are essential, and therefore these studies are challenging to perform under field conditions.

The main aim of our study was to investigate the clinical outcome and pathogen-shedding patterns of two pathogens, *C. parvum* and BCoV, in a comparative experimental mixed infection study, with the hypothesis that calves co-infected with *C. parvum* and BCoV would suffer a more severe acute phase and a more prolonged period of pathogen shedding than calves infected with either pathogen on its own. This study included comparing clinical signs (overall clinical score, fecal score, and weight gain) and pathogen-shedding patterns in calves naturally and experimentally infected with the pathogens.

## Materials and methods

### Overview of experimental design

**Figure 1:**
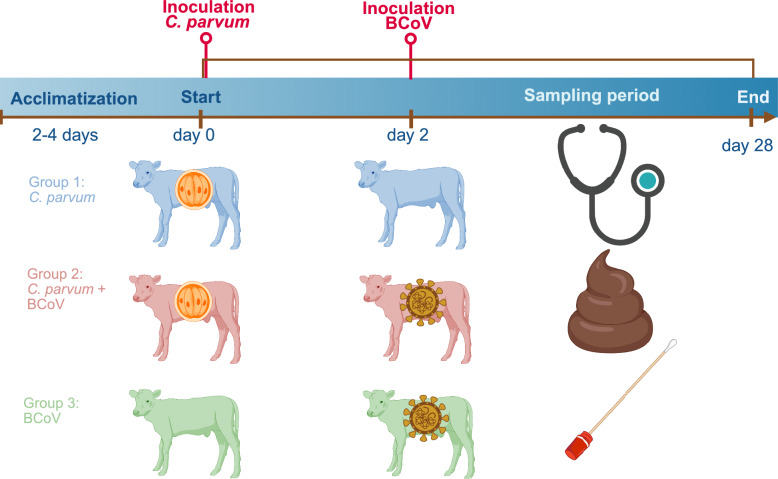
**Experimental design**. Created in BioRender. Varegg, M. (2026) https://BioRender.com/g3nb97h.

A comparative experimental infection study design was chosen. The study was conducted at the Moredun Research Institute (MRI), Scotland, using their experimental animal facilities. A total of 15 neonatal calves were included for a study period of 28 days from 1 day of age until euthanasia. Three experimental groups with five animals per group were established. The intervention was inoculation with *C. parvum* and/or BCoV. The first experimental group was inoculated with *C. parvum* only (G1). The second group with both *C. parvum* and BCoV (G2). The third group was inoculated with BCoV only (G3). Calves were acclimatized and inoculated (see “Infections” section) sequentially with *C. parvum* first and with BCoV 48 h later. The primary outcome was clinical signs, and secondary outcomes were the presence of fecal BCoV RNA and/or fecal *C. parvum* DNA. Clinical scoring and collection of fecal, nasal, and nasopharyngeal samples were conducted regularly throughout the study. An overview of the study design is shown in Figure 1.

Each experimental group was housed in separate animal study rooms. The calf groups were established and the experiment started successively as the calves arrived over a total period of 2 weeks. Hence, the calves were not randomized, as it was necessary for the calves within individual groups to be as close as possible in age. It was not possible to blind the researchers working with the calves, as the rooms needed to be entered in a certain order (group 1–3) to reduce the likelihood of contamination from one room to the next. When five calves had been allocated to a group, they were acclimatized for 2–4 days prior to inoculation.

### Calves

A resource equation [16] was applied to determine the minimum number of calves needed for this study, and 15 newborn calves of both sexes (10 females and 5 males) and mixed breeds were used in this study. The breeds were pure Holstein (*n* = 4), Holstein–British Blue mixes (*n* = 9), and Holstein–Aberdeen Angus mixes (*n* = 2). See Additional file 1 for an overview of the breeds and sex in each group.

All calves came from a single conventional British milk production farm and were transported to the research site within 12 h postpartum. The farm had not experienced recent problems with NCD. Calves were born naturally and fed colostrum from their dam at birth. The colostrum intake and quality were not monitored, as some calves suckled their dams immediately after birth. The dams were vaccinated with Bovigen^®^ Scour (Virbac Ltd., Bury St. Edmunds, UK) against bovine rotavirus, BCoV, and *E. coli* F5 (K99) prepartum. All calves were euthanized at the end of the experiment.

#### Housing and husbandry

At MRI’s livestock animal facilities, the study was performed in a separate accommodation from other animals. The experimental unit was each group of calves, housed in one individual room with a separate annex room where milk was prepared and scientists/veterinarians changed overalls, boot covers, gloves, and face masks prior to entering the animal accommodation. Calves were individually penned so that they could see each other but were not in direct contact with each other. Calves were housed on straw bedding with fresh straw added on top at least twice a week and more often if needed. At each feed calves were offered 2 L of milk replacer (“Creamy Calf”, Carrs Billington Agriculture Ltd., Carlisle, UK) prepared as described by the manufacturer’s instructions, morning and afternoon from a nipple bucket. The daily amount of milk replacer offered was increased by 1 L when calves were 5 days old and another 1 L daily when calves were 3 weeks old. Calves were offered water ad libitum from 1 week of age. They were not offered roughage or concentrates during the study.

#### Monitoring of calves

The calves’ behavior and demeanor were monitored in the morning and in the afternoon, in addition to a full clinical examination by a veterinarian once a day. If the calves showed signs of depression, an additional daily examination was performed.

#### Inclusion criteria

Only healthy, vital, newborn calves born from the herd of origin were included in the study. There were no other exclusion criteria defined prior to the start of the study, as the calves were enrolled almost immediately after birth.

### Infections

#### Inoculum

##### *Cryptosporidium parvum* inoculum

*Cryptosporidium parvum* oocysts from the Iowa isolate were purchased (*Cryptosporidium* Production Laboratory, University of Arizona, Tucson, AZ, USA). The viability of the oocysts was assessed by in vitro excystation of the stock prior to inoculation [17]. Briefly, a portion of the oocysts was transferred to an Eppendorf tube and centrifuged at 12 500 × *g* for 30 s. Supernatant was discarded, and 40 µL of Hanks Buffered Salt Solution (HBSS; prepared in-house) and 50 µL of 1% trypsin (VWR, Lutterworth, UK) prepared in HBSS at pH 3 was mixed with the pellet and placed in a 37 °C water bath for 1 h. After incubation, the Eppendorf tube was centrifuged as described above, and the supernatant was discarded. A total of 90 µL of HBSS, 10 µL of 2.2% sodium bicarbonate (Sigma, Gillingham, UK), and 10 µL of 1% sodium deoxycholate (VWR, Lutterworth, UK) in HBSS was mixed with the pellet and the Eppendorf was incubated in the water bath at 37 °C for 40 min. After incubation, the Eppendorf was centrifuged as described above, the supernatant discarded, and the pellet resuspended in 50 µL of phosphate buffered saline (PBS; prepared in-house) and mounted on a microscope slide with a cover slip. Oocysts (intact and unexcysted), oocyst shells, and sporozoites were counted under 400× magnification until a combined total of 250 was counted. Sporozoite ratio and excystation percentage were calculated as follows.

$${\mathrm{Sporozoite}}\,{\text{ ratio}} = \frac{{{\mathrm{Sporozoite}}\,{\mathrm{count}}}}{{{\mathrm{Shell}}\,{\mathrm{count}}}}$$

$${\mathrm{Excystation}}\,{\mathrm{rate}} = \frac{{{\mathrm{Shell}}\,{\text{ count}}}}{{\left( {{\mathrm{Oocyst}}\,{\mathrm{count}} + {\mathrm{Shell}}\,{\mathrm{count}}} \right)}} \times 100\%$$

The excystation rate of the batch of oocysts used for inoculation was 95.2%, and the sporozoite ratio was 3.1.

##### Bovine coronavirus inoculum

Fecal material was collected from an outbreak of winter dysentery in Norway in 2011 [18] and had been frozen at −80 °C at the Norwegian School of Veterinary Science since the outbreak. RNA was extracted from the fecal material and analyzed by quantitative polymerase chain reaction with reverse transcription (RT–qPCR) to determine the number of viral particles present. Briefly, RNA was extracted from 100 mg of feces with a commercial kit; RNeasy Mini Kit (Qiagen, Hilden, Germany), according to the to manufacturer’s description. Primers, probe, and cycling conditions for RT–qPCR are described in the “Quantification of *Cryptosporidium* and BCoV nucleic acids” section. The fecal material had a *C*_q_-value of 22.94. The fecal material had been analyzed by enzyme-linked immunosorbent assay (ELISA) for *E. coli* F5, bovine rotavirus, and *C. parvum* by the Diagnostic Department of The Norwegian Veterinary Institute and found negative for all the above. The inoculum was prepared by suspending 50 mL of fecal material in 50 mL of PBS, vortexing, and centrifuging at 500 *g* for 3 min at 4 °C. The supernatant was collected and used for intranasal inoculation. The pellet was resuspended in another 50 mL of PBS, vortexed, and centrifuged at 500 × *g* for 3 min at 4 °C, and the resulting supernatant was used for oral inoculation.

#### Inoculation procedure

Calves in G1 and G2 were inoculated orally with 1 × 10^6^ *C. parvum* oocysts, suspended in 5 mL PBS, on the first day of the experiment (day 0). Calves in G3 were mock-infected with 5 mL of PBS on the first day of the experiment.

Calves in groups 2 and 3 were inoculated intranasally and orally with 5 mL of fecal material containing BCoV 48-h after inoculation with *C. parvum*. Calves in group 1 were mock-infected as described above with PBS instead of inoculum.

### Clinical outcomes

#### Clinical score

Once daily, a full clinical examination was performed on each calf by a veterinarian. Some of the information from the clinical examination was used in a scoring system of the general and gastrointestinal signs. This scoring was developed on the basis of previous published studies [19–22] and is presented in Additional file 2. Calves with an overall clinical score > 3 were classified as “sick.” Clinical findings, other than those included in the scoring system, were also recorded.

#### Weight

All calves were weighed on arrival, and then once a week on the second, third, and fourth week of the study. They were weighed in a Ritchie 345G calf weighing crate (Oakleyweigh, Aylesbury, UK), which was disinfected with 3% hydrogen peroxide (Thermo Fisher, Hampton, USA) in between rooms.

#### Respiratory signs

The common respiratory signs for BCoV—respiratory rate, nasal discharge, and coughing—were monitored for all calves. Calves were considered to have an increased respiratory rate at ≥ 50 breaths/min; abnormal nasal discharge, if it was mucous to purulent; and coughing, if they were spontaneously coughing during the examination period of each room [21]. The examination period lasted approximately 30 min for each experimental room and was not performed in relation to feeding.

### Euthanasia

At the end of the study, the calves were humanely euthanized with a lethal dose of pentobarbital (Pentoject 200 mg/mL, Animalcare, York, UK) intravenously. Necropsy was performed at euthanasia.

### Humane end and intervention points

Humane end and intervention points were described prior to the experiment, to define when interventions would take place to reduce animal suffering.

Calves with an appetite score of ≥ 3 for more than two consecutive meals would be given additional electrolytes (Lectade Plus, Elanco Animal Health, Hook, UK) orally. Calves with an appetite score of ≥ 4 would additionally be tube-fed their meals until they started drinking independently from a bottle or nipple bucket again. Calves with severe diarrhea (fecal score ≥ 4) together with a behavior score of ≥ 3 and hydration score of ≥ 4 would be rehydrated intravenously according to standard veterinary practice in the UK. Calves with an abdominal pain score of ≥ 4 would be given nonsteroidal analgesic drugs according to standard veterinary practice in the UK.

Calves would be euthanized for animal welfare reasons if any of the following criteria occurred: calves were recumbent, severely depressed, and not able to reach food/water by themselves for over 12 h; calves showed signs of severe pain despite analgesic treatment; or other acute, unforeseen events occurred that led to severe impairment in calf welfare that could not be resolved by treatment.

Other incidents would be assessed by veterinarians on site and treated according to standard veterinary practice in the UK.

### Collection of material

#### Feces

Feces were collected daily from arrival to euthanasia for all the calves. Feces were taken directly per rectum, during defecation, or, as calves were individually stalled, fresh feces were picked up from the floor of the pen. Occasionally, it was not possible to procure a daily fecal sample from all the calves. A portion of the feces was transferred into 2 mL Eppendorf tubes and frozen at −20 °C until further analysis.

#### Nasal swabs

Nasal swabs were collected daily from all calves. A sterile, dry swab (Alpha laboratories, #SW1010, Eastleigh, UK) was inserted 3–5 cm in one nostril and rotated two to three times. Following swabbing, the handle was snapped off and the swab was placed into a 1.5 mL Eppendorf tube containing 1 mL of RLT buffer (Qiagen) and frozen at −70 °C until further analysis.

#### Nasopharyngeal swabs

Nasopharyngeal swabs were collected twice weekly from all calves. A soft nasopharyngeal tube containing a bristle swab was inserted into one nostril and advanced into the nasal passage until reaching the nasopharynx. The swab was extruded from the tube and rotated three times in the nasopharynx to collect the sample. The swab was withdrawn back into the tube before removing the tube from the nasal passage of the calf. The bristle swab was flushed in a 1.5 mL Eppendorf containing 1 mL of RLT buffer (Qiagen); remaining liquid was squeezed out from the bristle by applying pressure to the tube. The sample was frozen at −70 °C until further analysis.

### Laboratory analyses

#### Extraction of nucleic acids from feces

DNA and RNA were simultaneously extracted from fecal samples using Allprep Powerfecal Pro DNA/RNA kit (QIAgen). Briefly, fecal samples were thawed at room temperature and homogenized by stirring. Approximately 100 mg of fecal material was measured out for each extraction. The extraction was performed according to the manufacturer’s instructions, and DNA and RNA were each eluted in 100 µL of RNAse-free water. The concentration and quality of RNA and DNA were measured by photospectrometry on Multiskan Sky (Thermo Fisher Scientific). DNA and RNA from fecal samples were stored at −80 °C until further analysis.

#### Extraction of RNA from nasal and nasopharyngeal samples

RNA was extracted from nasal and nasopharyngeal samples by RNeasy Mini Kit (QIAgen). Briefly, samples were thawed on ice and homogenized by vortexing. A total of 200 µL of sample was mixed with 4 µL of 2 M dithiothreitol (DTT) and the extraction was performed according to the manufacturer’s instructions. RNA was eluted in 50 µL of RNAse-free water and the concentration and quality of RNA were measured by photospectrometry on Multiskan Sky before freezing at −80 °C until further analysis.

#### Quantification of *Cryptosporidium* and BCoV nucleic acids

Information on primers, probes, and cycling conditions for the (RT)-qPCRs is provided in Additional file 3. For quantification of *Cryptosporidium* DNA, the reaction mix was 20 µL, containing 10 µL KiCqstart Probe qPCR ReadyMix, Low ROX (Sigma Aldrich, St. Louis, USA), 2 µL PrimeTime qPCR Probe Assay (Integrated DNA Technologies, Coralville, USA), 3 µL of nuclease-free water (Thermo Fisher Scientific), and 5 µL of DNA template. For quantification of bovine coronavirus RNA, the reaction mix was 20 µL, containing 5 µL Taqman Fast Virus 1-step Master Mix for RT–qPCR (Thermo Fisher Scientific), 0.4 µL forward primer, 0.4 µL reverse primer, 0.8 µL probe, 11.4 µL nuclease-free water (Thermo Fisher Scientific), and 2 µL of RNA template. The (RT-)qPCRs were run in duplicates for each sample.

Positive controls for quantification of *C. parvum* were prepared as follows. DNA was extracted from 1 × 10^7^ *C. parvum* oocysts (Iowa II isolate, Bunch Grass Farm, Deary, Idaho, USA) with DNeasy Blood and Tissue Kit (QIAgen) as per the manufacturer’s description. A standard curve was prepared with tenfold serial dilutions of the DNA from 1 × 10^6^ to 1 × 10^1^ oocyst equivalents. Positive controls for quantification of BCoV were prepared as follows. A BCoV strain was maintained in an HRT-18G cell line. A cytopathic effect assay was used to determine the TCID_50_ of the strain [15]. RNA from 1 × 10^7^ TCID_50_ viral particles were extracted using a commercial kit (RNeasy, QIAgen), according to the manufacturer’s description. A standard curve was prepared with tenfold serial dilutions of the RNA from 1 × 10^7^ to 1 × 10^1^ TCID_50_ equivalents. Nuclease-free water was used as negative control.

Quantification was performed by extrapolating from the standard curves described above. Parasite and viral load in samples are expressed as log_10_ oocyst equivalents per reaction or log_10_ TCID_50_ equivalents per reaction, respectively. The cut-off for positive samples was set at <40 and <38 *C*_q_-values for the *Cryptosporidium* qPCR and BCoV RT–qPCR assays, respectively. Negative samples were therefore given a *C*_q_-value, which was extrapolated to a log_10_-value on the basis of the standard curve included in each assay. The negative threshold was 1.22 log_10_ oocysts and 1.51 log_10_ TCID_50_ for fecal samples, and 0.53 log_10_ and 0.55 log_10_ TCID_50_ for nasal and nasopharyngeal samples, respectively.

#### Subtyping of *C. parvum* samples

Fecal DNA samples taken on day 0, prior to any intervention, were analyzed for *C. parvum* subtypes on the basis of the gp60 gene. All gene products mentioned (SSU and gp60) should be in upright (not italics). A nested polymerase chain reaction (PCR) was performed on the basis of previous studies [23, 24]. Primers and cycling conditions are described in Additional file 3. Each reaction mix for the first PCR consisted of 25 µL of 12.5 µL DreamTaq PCR Master Mix (2×) (Thermo Fisher Scientific), 1 µL of each forward and reverse primers, 5.5 µL nuclease-free water, and 5 µL of DNA template. Each reaction mix for the second PCR consisted of 12.5 µL DreamTaq PCR Master Mix (2×), 1 µL of each forward and reverse primers, 8.5 µL of nuclease-free water, and 2 µL of the PCR product of the first PCR. Fecal DNA positive for *Cryptosporidium* on qPCR collected on days 0, 6, and 18 were also sequenced for species determination. A PCR-assay based on the SSU rRNA gene was performed [25]. Primers and cycling conditions are described in Additional file 3. Each reaction mix consisted of 25 µL of 12.5 µL DreamTaq PCR Master Mix (2×), 1 µL of each forward and reverse primers, 5.5 µL of nuclease-free water, and 5 µL of DNA template. After confirmation on a 1.5% agarose gel, PCR-positive samples were cleaned with ExoSAP-IT™ PCR Product Cleanup (Thermo Fisher Scientific) according to the manufacturer’s instructions. Briefly, 2 µL ExoSAP-IT reagent was mixed with 5 µL PCR-product and incubated at 37 °C for 15 min and then at 80 °C for 15 min. The cleaned product was sent for Sanger sequencing in both directions at a commercial facility (Eurofins, Konstanz, Germany). The genomic sequences were entered into the National Center for Biotechnology Information (NCBI) Basic Local Alignment Search Tool (BLAST) to confirm *Cryptosporidium* species, and for *gp60* subtype determination, the serine codons were counted manually [26].

#### Analysis of other infectious agents

Fecal samples taken from all calves prior to experimental inoculation were analyzed for common pathological agents known to cause NCD. An ELISA antigen test (Rota-Corona-k99 Ag Test, IDEXX, Hoofddorp, Netherlands) was performed. RNA was isolated from nasal swabs taken from calves on day 7 and analyzed in a multiplex qPCR for common viral and bacterial pathogens causing respiratory disease in calves (Pneumo 4BVC55, DNA Diagnostic, Risskov, Denmark).

### Statistical analyses

Results are presented as descriptive statistics using Microsoft Excel 2024 and BioRender software. Statistical analyses of differences in clinical outcomes or amount of *C. parvum* DNA and BCoV RNA between the three groups were analyzed by one-way analysis of variance (ANOVA) and Tukey Test for pairwise comparisons or by nonparametric Kruskal–Wallis and Dwass–Steel–Critchlow–Fligner (DSCF) pairwise comparisons in Jamovi 2.3.18. Statistical analyses of differences in *C. parvum* DNA or BCoV RNA from baseline levels to different days of the study within each group was analyzed by paired samples *t*-test in Jamovi 2.3.18.

## Results

### Exclusion of data from specific calves owing to other incidents

Two calves in G1 presented clinical signs in accordance with an eye infection and upper respiratory tract infection. The clinical signs were mild, and no intervention was needed. Clinical score parameters that were affected and related to the pathology of an eye and upper respiratory tract infection were excluded from the relevant time periods for each calf.

One calf in G1 showed clinical signs in accordance with an infection on the ear tag site on the last 3 days of the experiment. The infection was treated locally by cleaning the infected wound and administrating an antimicrobial spray (Terramycin Aerosol Spray, 3.92% w/w, Farm Vet Supplies, Roslea, UK) after consulting with the onsite veterinarians. Clinical score parameters that were affected and related to the pathology of an ear tag infection were excluded from day 24 until the end of the study for this calf.

### Humane end and intervention points

Two calves reached the predetermined intervention points. One calf in G1 was administered additional electrolytes (Lectade Plus, Elanco) on one occasion (experimental day 7). One calf in G2 was administered electrolytes and was tube-fed for two separate meals (day 4 and day 6).

### Infection outcomes and presence of other pathogens

Fecal samples from all calves were investigated for several pathogens on day 0, prior to *Cryptosporidium* infection. A total of 12 calves were found to be shedding *Cryptosporidium* DNA in their feces. Three calves in G1, four calves in G2, and five calves in G3 had detectable fecal *Cryptosporidium* DNA. This indicates that at least 12 of the calves were infected with *Cryptosporidium* on the farm where they were born, before they arrived at the research site.

Feces from all calves prior to infection were negative for bovine coronavirus, bovine rotavirus, and *Escherichia coli* antigen K99 by ELISA antigen test (IDEXX). Nasal swabs from all calves taken on day 7 were negative for bovine viral diarrhea virus, bovine herpesvirus 1, bovine respiratory syncytial virus, and bovine parainfluenza virus. A few calves were positive for common commensal bacteria found in the upper respiratory airways (*Mannheimia haemolytica*, *Pasteurella multocida*, *Mycoplasma bovis*, and *Histophilus somni*). In G1, one calf was positive for *M. haemolytica* and one calf positive for *H. somni*. In G2, one calf was positive for *P. multocida*, *M. haemolytica*, *M. bovis*, and *H. somni*. In G3, one calf was positive for *P. multocida*, *M. haemolytica*, *M. bovis*, and *H. somni*.

On day 0, prior to any experimental infection, there was no detectable BCoV RNA by RT–qPCR in any of the calves’ fecal, nasal, or nasopharyngeal samples. None of the G1 calves tested positive for BCoV RNA in fecal samples analyzed by RT–qPCR on days 0, 3, 5, 7, 9, 14, 21, or 28, indicating that there was no apparent contamination between the experimental units in this study.

As several calves had been naturally infected with *C. parvum* prior to arrival at the study site and experimental inoculation, the infection status of each group was adjusted accordingly (Table 1). G1 was therefore described as naturally and experimentally infected with *C. parvum*; G2 was naturally infected with *C. parvum* and experimentally infected with *C. parvum* and BCoV; and G3 was naturally infected with *C. parvum* and experimentally infected with BCoV.

**Table 1:** **Overview of natural and experimental infections**

	G1	G2	G3
*Planned experimental design*			
Experimental *C. parvum* infection	X	X	
Experimental BCoV infection		X	X
*Actual experimental design*			
Natural *C. parvum* infection	X	X	X
Experimental *C. parvum* infection	X	X	
Experimental BCoV infection		X	X
Infection status	Natural and experimental infection with *C. parvum*	Natural and experimental infection with *C. parvum*. Experimental infection with BCoV	Natural infection with *C. parvum*. Experimental infection with BCoV

Overview of the original experimental design and the adjusted infection status of each experimental unit due to natural infection of *C. parvum*.

### Clinical outcomes

#### Clinical scores

##### Overall clinical score

**Figure 2:**
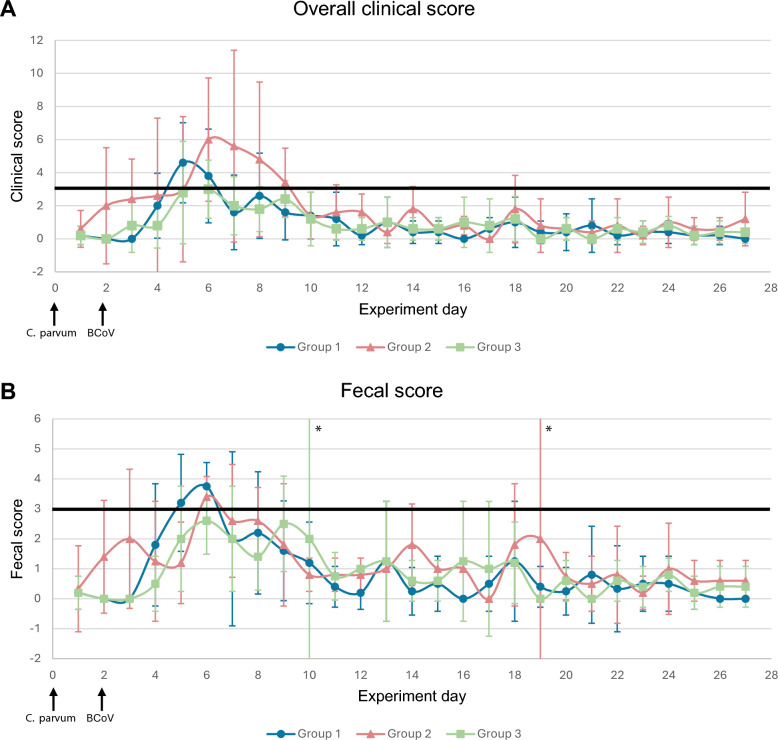
**Clinical and fecal score**. Mean clinical and fecal scores with 95% confidence interval of each group of calves on given days during the experiment. **A** Mean clinical score of groups of calves during the 28 days of the experiment. **B** Mean fecal score of groups of calves during the 28 days of the experiment. The black line in each graph shows the threshold of “disease.” Calves with a clinical score > 3 were classified as sick, and calves with a fecal score > 3 were classified as diarrheic. Group 1: natural and experimental infection with *C. parvum*. Group 2: natural and experimental infection with *C. parvum* and experimental infection with BCoV. Group 3: natural infection with *C. parvum* and experimental infection with BCoV. ^*^For these groups on these days, there were only two registrations, giving an unreliable foundation for calculating the confidence intervals.

In general, all calves showed mild clinical signs in this study, and the majority of clinical signs presented within the first 10 days of the study (Figure 2). Total days with an overall clinical score > 3 from all calves showed that they were “sick” for around 8% of the duration of the study (28 days). Mean overall clinical score from all calves reached the threshold of “sick” from day 5 to day 8, with the highest score on day 6 (score 4.23). All calves in G1 and G2 had at least 1 day classified as sick whereas 1/5 calves in G3 were classified as sick during the experiment (Additional file 4). The calves in G1 and G2 showed a tendency toward more clinical signs over a longer period of time (Figure 2). The calves in G1 and G2 had, respectively, 4 and 5.6 times more sick days than the calves in G3 over the entire duration of the experiment (Table 2), but the difference between groups by nonparametric Kruskal–Wallis test (*ε*^2^ = 0.424, *P* = 0.051) showed only a tendency toward significance. If only the first 10 days of the study are considered, then a significant difference in the proportion of days that the calves were sick between the groups was apparent (*η*^2^ = 0.425, *P* value: 0.036), with group comparisons G1–G2 (*P* = 0.89), G1–G3 (*P* = 0.092), and G2–G3 (*P* = 0.041). The mean overall clinical score was the highest for G1 on day 5 (score 4.6), and G1 exceeded the threshold of being “sick” on days 5 and 6. The mean overall clinical score was the highest for G2 on day 6 (score 6), and G2 exceeded the threshold of “sick” from days 6 to 9. The mean overall clinical score was the highest for G3 on day 6 (score 3) but did not reach the threshold to be classified as sick on any given day of the experiment.

**Table 2:** **Sum of days when groups of calves presented clinical signs**

Group	Peak rt (°C)	Number of days with						Peak clinical score	Days with clinical score > 3
		Increased rt	Depression	Diarrhea	Decreased appetite	Dehydration	Pain		
1	39.7	5	1	21	1	2	2	8	12
2	40.2	13	7	27	6	11	5	12	17
3	39.8	4	0	15	0	4	2	7	3

Sum of days all calves in each group presented clinical signs. Peak clinical score: the calf with the highest clinical score in the group on any given day in the study. Group 1: natural and experimental infection with *C. parvum*. Group 2: natural and experimental infection with *C. parvum* and experimental infection with BCoV. Group 3: natural infection with *C. parvum* and experimental infection with BCoV. *rt*, rectal temperature

##### Fecal score

Fecal score was the individual score that influenced the overall clinical score the most.

All calves in G1 and G2 had fecal scores classified as diarrhea (fecal score 3) and severe diarrhea (fecal score 4) during the experiment, whereas 4/5 and 1/5 calves in G3 had fecal scores classified as diarrhea and severe diarrhea, respectively (Additional file 4). The mean fecal score in G3 also never reached the threshold of diarrhea on any given day of the study (Figure 2). The proportion of days with diarrhea in all groups was similar (*P* value = 0.376) during the first 10 days of the study. For the proportion of days with severe diarrhea during the first 10 days of the study, the Kruskal–Wallis test did not show a significant difference (*P* value = 0.092) but a large effect size (*ε*^2^ = 0.341), suggesting meaningful group differences. Although the data are skewed for this effect (proportion of days with severe diarrhea), it is relevant to note that G3 only had one animal with positive values and could be an explanation for the data not being normally distributed. One-way ANOVA showed significant differences in severe diarrhea between groups (*η*^2^ = 0.463, *P* value = 0.024), and post hoc comparisons indicated a significant difference between G1 and G3 (*P* = 0.022).

#### Weight gain

Upon arrival at the research site, the weight of the calves varied from 36.4 to 53.0 kg, with the mean weight for all calves at 46.5 kg. At euthanasia, the weight varied from 44.6 to 62.6 kg, with the mean weight for all calves at 56.7 kg. The percentage weight gains for each group varied during the study. From arrival to euthanasia, the mean percentage weight gain (with 95% confidence interval [CI]) was 14.63% (11.29, 17.97), 22.23% (15.47, 29.00), and 30.15% (22.03, 38.28) for groups G1, G2, and G3, respectively, with a significant difference in weight gains between G1 and G3 (Additional file 5). In both G1 and G2, two calves lost weight from arrival to the second week of the study, and calves in G1 gained less weight in the first 3 weeks than calves in G2 and G3 (Additional file 5). From the third week and until euthanasia, all calves put on similar amounts of weight.

#### Respiratory signs

All calves presented with mild respiratory signs in this study. None of the calves showed an increased respiratory rate after inoculation with BCoV.

The total number of days that calves in each group had abnormal nasal discharge was 3, 15, and 24 days, in G1, G2, and G3, respectively. Four of the five calves in G2 and all five calves in G3 presented with abnormal nasal discharge during the study. The proportion of calves in G2 and G3 with abnormal nasal discharge was the highest on days 6, 8, and 9 post inoculation, where five, four, and six calves had abnormal nasal discharge, respectively.

The total number of day calves in each group were coughing was 0, 12, and 5 days, in G1, G2, and G3, respectively. In both G2 and G3, four of five calves presented with coughing during the study. The proportion of inoculated calves with cough was the highest 6–7 days and 21 days post inoculation, where two of ten calves were coughing.

#### Necropsy

Necropsy was performed on all calves after euthanasia. No specific macroscopic lesions were observed in the gastrointestinal or respiratory tract in any of the calves.

### Shedding of pathogens

#### Feces

##### *Cryptosporidium* detection

A total of 12 calves were positive for fecal *Cryptosporidium* DNA on day 0, prior to any infection (three calves in G1, four calves in G2, and five calves in G3). The positive calves shed a mean of 3.51 (95% CI: 3.12, 3.90) log_10_ oocyst per PCR reaction.

**Figure 3:**
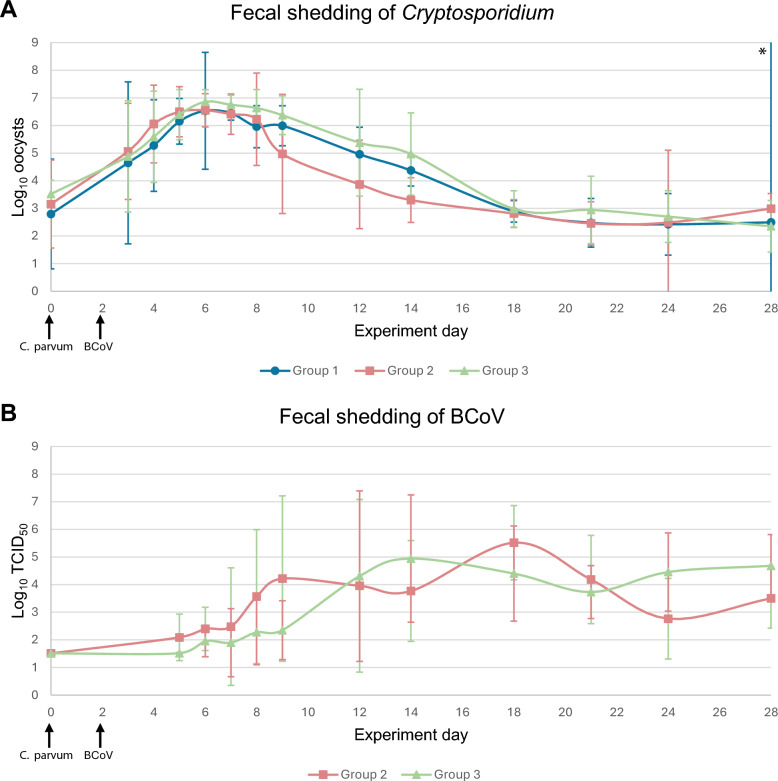
**Shedding of *Cryptosporidium* and BCoV from feces**. Mean log_10_ *Cryptosporidium* oocyst equivalents and BCoV TCID_50_ equivalents per PCR reaction with 95% confidence intervals in fecal samples of each group of calves on various days during the experiment. A standard curve of 10^1^–10^6^ oocyst equivalents was used to extrapolate calf sample *Cryptosporidium* data to log_10_ values. A standard curve of 10^1^–10^7^ TCID_50_ equivalents was used to extrapolate calf sample BCoV data to log_10_ values. Samples taken on day 0 are pre-inoculation samples, serving as a baseline for each group. The negative threshold for *Cryptosporidium* and BCoV was 1.22 log_10_ oocyst equivalents and 1.51 log_10_ TCID_50_ equivalents, respectively. **A** Mean log_10_ *Cryptosporidium* oocysts in groups of calves during the 28 days of the experiment. **B** Mean log_10_ BCoV TCID_50_ in groups of calves during the 28 days of the experiment. ^*^For this group on this day, there were only two registrations, giving an unreliable foundation for calculating the confidence interval.

**Figure 4:**
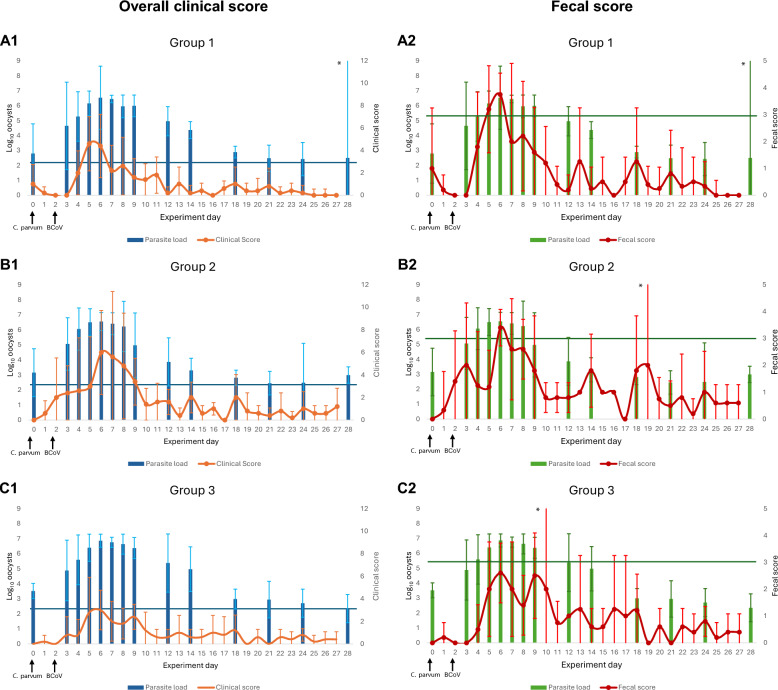
**Shedding of *Cryptosporidium* and clinical outcomes**. Associations between clinical signs and presence of *Cryptosporidium* DNA in feces. The horizontal lines represent the threshold when a clinical score is classified as sick and a fecal score is classified as diarrheic. **A1** Association between clinical scores and fecal shedding of *C. parvum* in G1. **A2** Association between fecal scores and fecal shedding of *C. parvum* in G1. **B1** Association between clinical scores and fecal shedding of *C. parvum* in G2. **B2** Association between fecal scores and fecal shedding of *C. parvum* in G2. **C1** Association between clinical scores and fecal shedding of *C. parvum* in G3. **C2** Association between fecal scores and fecal shedding of *C. parvum* in G3. ^*^For these groups on these days, there were only two registrations, giving an unreliable foundation for calculating the confidence intervals.

The shedding pattern of *Cryptosporidium* was similar in all groups of calves (Figure 3). By day 3, all calves in G2 and G3, and by day 4, all calves in G1 shed fecal *Cryptosporidium* DNA. All groups of calves were increasingly shedding oocysts, peaking on day 6 with means of 6.53 (4.42, 8.65), 6.55 (5.95, 7.40), and 6.86 (6.42, 7.30) log_10_ oocysts per PCR reaction in G1, G2, and G3, respectively. By day 5, all groups of calves had a statistically significant increase in *Cryptosporidium* DNA from baseline levels (*P* = 0.032, 0.019, and 0.003 for G1, G2, and G3, respectively). The days the calves were shedding the most *Cryptosporidium* DNA correlated with the days calves presented the most gastrointestinal and general signs (Figure 4).

From peak shedding day, calves shed decreasingly less *Cryptosporidium* DNA until the end of the experiment, with a mean from all calves of 2.61 (2.17, 3.05) log_10_ oocysts on day 28, and below the baseline levels from day 0. Fecal shedding of *Cryptosporidium* in G2 showed a tendency toward a more rapid decline than in the other two groups, with at least one log_10_ value difference in means of oocysts between G2–G1 and G2–G3 on days 9, 12, and 14. There was a significant difference between all groups on day 14 (*η*^2^ = 0.477, *P* = 0.028), and by pairwise comparisons, G2 differed from G3 (*P* = 0.024). There were no significant differences between the groups on day 9 and 12. It was also noted that among some calves with very few (or zero) days with diarrhea, high shedding of *Cryptosporidium* DNA was recorded (data not shown).

##### Bovine coronavirus

All calves were negative for fecal BCoV RNA prior to any infections (day 0), and G1 stayed negative for fecal BCoV RNA throughout the experiment. In total, 3 days after inoculation with BCoV (day 5), BCoV RNA was detected in fecal samples of four calves in G2 and 1 calf in G3. By day 7, all calves in G2, and by day 14, all calves in G3 shed fecal BCoV RNA (Figure 3).

**Figure 5:**
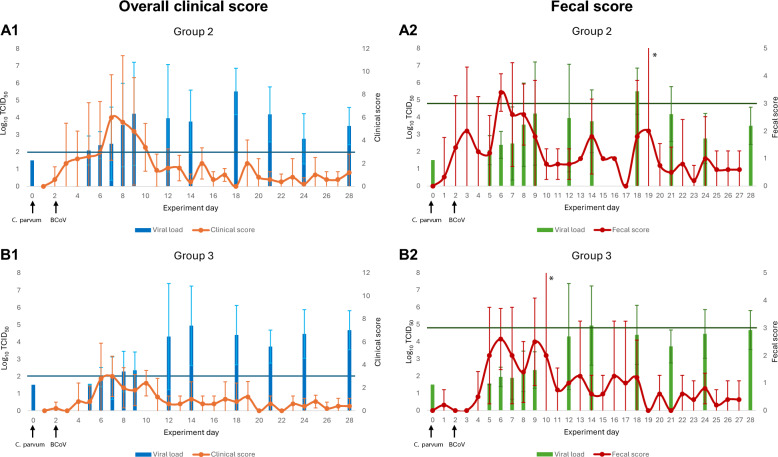
**Shedding of BCoV and clinical outcomes**. Association between clinical signs and presence of BCoV RNA in feces. The horizontal lines represent the threshold when a clinical score is classified as sick and a fecal score is classified as diarrheic. **A1** Association between clinical scores and fecal shedding of BCoV in G2. **A2** Association between fecal scores and fecal shedding of BCoV in G2. **B1** Association between clinical scores and fecal shedding of BCoV in G3. **B2** Association between fecal scores and fecal shedding of BCoV in G3. ^*^For these groups on these days, there were only two registrations, giving an unreliable foundation for calculating the confidence intervals.

The days calves infected with BCoV (G2 and G3) presented the most clinical signs were in the initial phase of BCoV RNA fecal shedding (Figure 5). Shedding of fecal BCoV RNA started to increase on day 5 (G2) and day 6 (G3) from baseline levels, with the groups presenting most clinical signs between days 6 and 9 (G2) and on day 6 (G3). However, a significant increase in mean BCoV RNA from baseline levels did not occur until day 14 in both groups (G2, *P* = 0.026; G3 *P* = 0.014), and they continued to shed fecal BCoV RNA over baseline levels until the end of the study. During this time period, the calves presented few to no clinical signs. There were no statistically significant difference between G2 and G3 on any day analyzed for presence of fecal BCoV RNA.

##### Species and subtype determination

The fecal DNA sequenced for the SSU gene was successful for one sample on day 0, eleven samples on day 6, and none of the samples taken on day 18. All PCR-positive samples demonstrated *C. parvum*.

The fecal DNA sequenced for the *gp60* gene was successful for four samples on day 0 (prior to inoculation), and two different subtypes were determined; IIaA17G2R1 and IIaA18G2R1, present in G1 and G2. The samples with the highest load of fecal *Cryptosporidium* DNA were subtyped on later collection days (days 4–7) and determined as IIaA17G2R1, IIaA18G2R1, and IIaA19G2R1. From all samples subtyped, G1 had the presence of only subtype IIaA17G2R1; G2 had subtypes IIaA17G2R1 and IIaA18G2R1; and G3 had subtypes IIaA18G2R1 and IIaA19G2R1. By the time we sent samples for sequencing, there was no inoculum left from the infection experiment and so the inoculum was not subtyped. We subtyped the positive control used for confirming PCR-positive samples on agarose gel, which was an Iowa isolate from Bunch Grass Farms (Deary, ID, USA). The positive control was subtyped as IIa17G2R1.

#### Nasal and nasopharyngeal swabs

Prior to any intervention, all calves were negative for nasal and nasopharyngeal BCoV RNA. By day 5 of the experiment (3 days after inoculation), BCoV RNA was detected in nasal swabs and nasopharyngeal swabs in nine of ten calves (G2 and G3). By day 12 of the experiment, all calves (G2 and G3) were positive for nasal and nasopharyngeal BCoV RNA. Nasal swabs from G1 were analyzed on day 7 and were negative for BCoV RNA.

**Figure 6:**
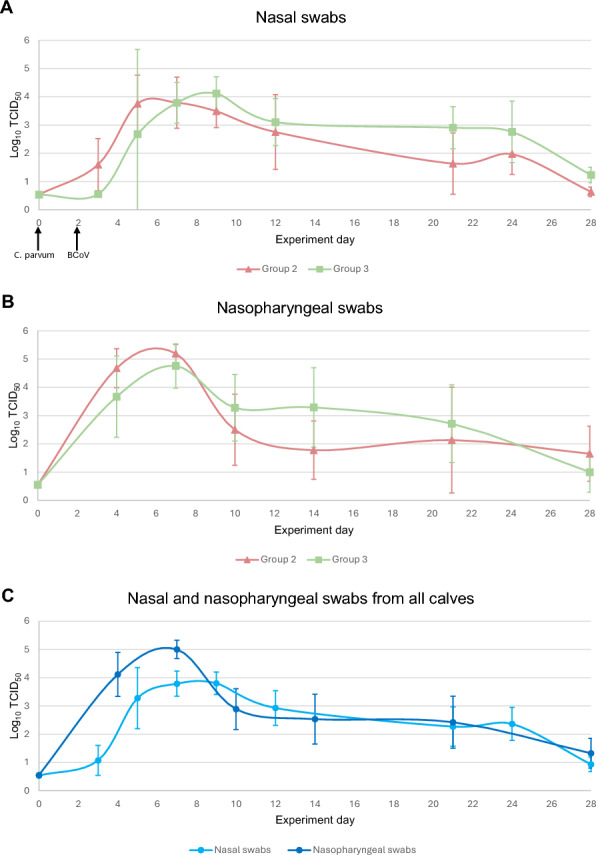
**Shedding of BCoV from respiratory tract**. Mean log_10_ BCoV TCID_50_ equivalents per qPCR reaction with standard deviation in nasal and nasopharyngeal swabs of calves in groups 2 and 3 on given days during the experiment. A standard curve of 10^1^–10^7^ TCID_50_ equivalents of BCoV was used to extrapolate calf sample data to log values. Samples taken on day 0 are pre-inoculation, serving as a baseline for each group. The negative threshold had was 0.53 and 0.55 log_10_ TCID_50_ equivalents for nasal and nasopharyngeal samples, respectively. **A** Mean log_10_ BCoV TCID_50_ in nasal swabs from G2 and G3 during the 28 days of the experiment. **B** Mean log_10_ BCoV TCID_50_ in nasopharyngeal swabs from G2 and G3 during the 28 days of the experiment. **C** Mean log_10_ BCoV TCID_50_ in nasal and nasopharyngeal swabs from all animals (combined G2 and G3) during the 28 days of the experiment.

The amount and distribution of both nasal and nasopharyngeal shedding of BCoV RNA were similar in both inoculated groups (Figure 6). Both groups were increasingly shedding nasal BCoV RNA until peak shedding day 7 (G2) and day 9 (G3). Similarly, both groups have peak shedding of nasopharyngeal BCoV RNA on day 7. Shedding of BCoV from the upper respiratory tract declined until day 28. All calves had higher detectable number of BCoV RNA in the nasopharyngeal samples than the nasal samples.

The peak shedding days for nasal and nasopharyngeal BCoV RNA correlates with the days that most calves presented with clinical signs from the upper respiratory tract. The proportion of calves with abnormal nasal discharge and coughing was high between days 6 and 9 in groups inoculated with BCoV, and the shedding from nasal swabs peaks on days 7 (G2) and 9 (G3) and from nasopharyngeal swabs on day 7 (G2 and G3).

## Discussion

This comparative experimental study conducted at MRI research facilities presents clinical outcomes and pathogen shedding patterns in neonatal calves inoculated with *C. parvum*, BCoV, or both pathogens. In addition, the majority of the calves had a natural infection of *C. parvum*. We found that naturally infected calves with additional experimental *C. parvum* infection presented more clinical signs over a longer time period than calves only naturally infected with *C. parvum*, despite these also being co-infected with BCoV. However, the shedding pattern of *Cryptosporidium* is similar in all groups despite the difference in clinical outcomes. Our findings also highlight the difficulties procuring *Cryptosporidium*-free calves for experimental purposes.

The sample size of our study was small, which could result in reduced statistical power and bias owing to individual variations influencing the results. Therefore, extrapolation to field conditions should be done with care. Controlled conditions are imperative when investigating the clinical impacts on neonatal calves that are associated with commonly occurring enteropathogens, and confounding factors that might occur under field conditions, like other mixed infections and herd effects, should be minimized. Systematic and repeated sampling over time provides a more accurate description of infection dynamics, such as onset, duration, cessation, and associations with clinical signs, than “situation snapshots” that are usually described in field studies.

### Natural and experimental infection with *C. parvum *seems to exacerbate clinical signs compared with a mixed infection with *C. parvum* and BCoV

Our findings suggest that experimental and natural infection with *C. parvum* exacerbates clinical signs and diarrhea in calves, compared with calves co-infected with *C. parvum* and BCoV. In the case of overall clinical score, calves with both natural and experimental *C. parvum* infection, together with BCoV infection (G2), had the highest proportion of “sick” days, which was significantly greater than the calves with BCoV and natural *C. parvum* infection (G3). Similarly, the calves with both natural and experimental *C. parvum* infection (G1) had the highest proportion of days with severe diarrhea, which was significantly greater than the calves with BCoV and natural *C. parvum* infection (G3). Together, these results indicate that the combined load of the natural and experimental *C. parvum* infections was more likely to result in clinical signs and diarrhea than the natural *C. parvum* and experimental BCoV co-infection. This tendency has also been reported in co-infection studies with *C. parvum* in lambs, where the animals developed serious clinical illness, independent of whether they were infected with *C. parvum* alone or co-infected with *C. parvum* and enterotoxigenic *E. coli* (ETEC) or rotavirus [27]. However, our results contrast with those of a study investigating NCD in New Zealand dairy farms in which the likelihood of calves having liquid feces was significantly higher on the farms where co-infections were present than in farms with mono-infections or absence of infections [28]. Furthermore, in an outbreak of NCD on a dairy farm in China, watery diarrhea was associated with co-infections with *C. parvum* and rotavirus [29]. Similarly, an experimental study with *C. parvum* and rotavirus in piglets reported a higher proportion of days with diarrhea and oocyst shedding, as well as decreased body weight gain, in animals co-infected with both agents compared with piglets only infected with *C. parvum* [13]. This suggests a disparity between mixed infections of different origins but may also reflect other differences such as time of infection, immune status, and infectious dose. Although literature indicates exacerbation of clinical signs during co-infection with *C. parvum* and rotavirus, our findings suggest that the double natural and experimental infection with *Cryptosporidium* is more likely to result in clinical illness than *C. parvum* and BCoV together.

This disparity is also reflected in our results on associations between pathogen shedding and clinical outcomes, where peak clinical outcomes and shedding of *C. parvum* occurs in the same time period, whereas peak shedding of BCoV occurs after cessation of most clinical signs. However, previous investigations on calves naturally and experimentally challenged with BCoV report a good correlation between clinical score and detection of BCoV RNA in feces [21]. It is likely that the BCoV infection in our study produced mild clinical outcome owing to calves receiving maternal colostrum from dams vaccinated against BCoV. It has been shown that dry-cow vaccination significantly boosts calves’ antibody titers against BCoV and can positively affect the clinical outcome and detection of pathogens [30]. This effect may have amplified the relative clinical importance of *C. parvum* infection compared with BCoV infection in our study. However, obtaining calves fed colostrum from their natural dams was done purposely in our study to mimic field conditions. Vaccination of dams prepartum against common pathogens causing NCD (BCoV, BRV, and *E. coli*) is performed in industrialized farming [31], and bulk tank milk in dairy farms is commonly found to be seropositive for BCoV in Europe [32]. Also, a longitudinal study following beef calves reported that during an outbreak of bovine respiratory disease associated with BCoV shedding, levels of anti-BCoV antibodies were not associated with disease incidence [33].

We found it surprising that the calves only infected with *C. parvum* had the lowest weight gain in the study, especially considering the calf breeds in each group. Both G2 (1) and G3 (3) had pure Holstein calves present in the group, whereas all the calves in G1 were beef crosses. We expected that beef-crossed calves would have a weight-gain advantage over their pure-bred dairy peers [34] but that was not the case in this study. Calves of both G1 and G2 had diarrhea and general clinical signs over more days than the calves in G3, but the calves in G1 had the most days of severe diarrhea (fecal score 4), which could explain the differences in weight gain between the groups.

In vitro studies investigating interactions between *C. parvum*, BCoV, and host cells in single- and co-infected HCT-8 cell lines report that 1 h after infection, the amount of BCoV RNA present is higher in cells co-infected with *C. parvum* [15], hypothesizing that the parasite aids the virus entry into cells but without further effects on viral replication. By using the same in vitro system, gene expression profiling of the HCT-8 cells showed that BCoV influenced the host-cell response to a higher degree than *C. parvum* in co-infected cells [35], but infection rates for BCoV were higher than for *C. parvum*, which could have biased the results. In our in vivo study, we observe that the double infection (natural and experimental) with *C. parvum* results in a worse clinical outcome than a mixed infection with BCoV, suggesting that results from in vitro models cannot always be extrapolated to more complex, living organisms.

### An “uncontrolled” natural infection in addition to the experimental inoculation does not seem to affect the shedding pattern in challenged calves

The calves in our study had an “uncontrolled” natural *C. parvum* infection, where most of the calves, including those not subsequently challenged with oocysts, were shedding *C. parvum* DNA prior to inoculation. A study investigating the dose–response relationship in *C. parvum*-infected calves observed no significant correlation between inoculation dose and magnitude and duration of oocyst shedding [36]. We observed a similar trend in our calves, where despite the dose being uncontrolled in the natural infections, all calves had similar shedding patterns regarding duration and peak of shedding. These findings emphasize the difficulties in managing cryptosporidiosis outbreaks, particularly as we observed that several of the animals with few to no days of diarrhea were some of the highest shedders of *C. parvum* DNA on some days throughout the study.

However, it is important to note that in our study, we did not collect the total fecal output of the calves. One daily sample from each calf is not necessarily representative of the total load of oocysts shed throughout the day, and it has been shown that feces from calves sampled twice a day can vary in oocyst numbers [37]. Nevertheless, a study comparing oocyst numbers in interval collection and complete fecal collection did not observe any statistical difference in the two sampling methods or between morning and evening samples in the interval collections in *C. parvum*-challenged calves [38]. Although literature is ambiguous, it is possible that we have missed some important variations in the calves by only including one fecal sampling a day. In addition, we did not measure oocysts per dry matter of feces and diarrheic feces will dilute the amount of *C. parvum* DNA detected. Considering there is a significant difference in diarrhea severity between groups, it is possible that the oocyst shedding peaks in the diarrheic calves were underestimated in our study. Studies should consider increasing the sampling intervals to more than once daily and attempt to relate oocyst counts to fecal dry matter to avoid confounding results.

### Challenges performing *C. parvum* experimental studies with neonatal calves

The majority of the calves in our study were positive for *C. parvum* prior to infection, despite transporting them to the farm within 12 h of birth. Of the samples that were possible to sequence, similar *gp60* subtypes were found, so it is possible that the strain they were inoculated with had the same *gp60* subtype as one of the natural infections from the farm of origin. However, analysis of the *gp60* gene may not provide enough resolution to discriminate between similar subtypes, as it only compares the variation in a single locus. For more in-depth differentiation of the subtypes, analysis of several loci (e.g., a multilocus variable number tandem repeat analysis [39]) could provide more granular information.

Early infection and shedding of *Cryptosporidium* have previously been reported from Sweden, where a 1-day-old beef calf was shedding *C. bovis* oocysts [40], and in the UK, where calves sampled from 1 to 2 days of age were shedding *C. parvum* oocysts [41]. In vivo experiments on production animals are often forced to rely on obtaining appropriate animals from commercial farms where *Cryptosporidium* is prevalent and difficult to control [7]. The oocysts are environmentally stable and resistant to most common disinfectants, and there is no treatment available that completely eliminates the oocyst shedding in cattle [42–44]. In addition, a low dose is needed for infection in calves, whereas a calf can shed billions of oocysts daily [36], without necessarily showing any clinical signs [45]. These factors, together with how early neonatal calves can become infected with *C. parvum*, highlight the challenges of procuring *Cryptosporidium*-free calves for animal experimentation. Some methods to mitigate natural *C. parvum* infection in neonatal calves employed in animal experiments have been described. For example, delivering the calf on a clean surface (e.g., covered in plastic), so that the calf does not come in contact with the farm environment, has been used in studies [36, 38]. This is, however, time-consuming and requires trained personnel to be available around the clock and could also contribute to a prolonged period of procuring the calves for participation in experiments. Another strategy is to use genetically modified *C. parvum* that is resistant to commonly used drugs (e.g., paromomycin) for the experimental infections and “treat away” the natural infection. This approach has been used in a recently published study, looking at *C. parvum* intestinal infection sites in the calf and mouse model [46]. However, although paromomycin was efficient in most calves, there is no perfect drug for completely eliminating *C. parvum*, and the study found that the tissues from a couple of animals in the negative control group were still positive for *C. parvum* by qPCR but negative for the genetically modified challenge parasite, indicating that the parasite had been contracted on the farm of origin [46].

### BCoV causes both respiratory and gastrointestinal clinical signs in young calves

Although BCoV is established as a causative agent for diarrhea in young calves, there has been ambiguity in literature regarding BCoV’s role in respiratory disease in calves and a hypothesis that BCoV that infect the gastrointestinal tract or respiratory tract differ antigenically and genomically and belong to two different groups [47]. However, studies on BCoV performed in experimental settings show that calves present clinical signs from both the gastrointestinal and respiratory tracts. Seronegative calves co-mingled with clinically sick calves from a winter dysentery outbreak developed respiratory signs although the origin of infection was from a gastrointestinal outbreak [21]. And calves experimentally infected with a BCoV strain (MN-1988) maintained on HRT-18 cells produced both gastrointestinal and respiratory signs in young calves [48]. Another study focusing on route of infection observed that both intranasal and oral infection with BCoV resulted in both respiratory and gastrointestinal sign in calves [49]. Similarly, the calves in our study infected with fecal material from an outbreak of winter dysentery showed mild respiratory disease, which correlated well with shedding patterns detected by nasal and nasopharyngeal swabs. Interestingly, shedding of fecal BCoV did not seem to correlate well with the calves’ fecal score in our study, although the inoculation strain came from an enteric outbreak. The calves in our study were also younger than in the two other studies, suggesting that BCoV could be associated with mild respiratory signs in neonatal calves.

There was also a tendency that the calves, which were infected both experimentally and naturally with *C. parvum* in addition to co-infection with BCoV, started to shed viral particles in both feces and samples from the upper respiratory tract earlier than the calves naturally infected with *C. parvum* and co-infected with BCoV. A study investigating the effect of bovine rotavirus, BCoV, and *C. parvum* on calf health reported that calves positive for *C. parvum* had a higher chance of being treated against respiratory disease [12] and theorizes that *C. parvum* causes immunosuppression predisposing to respiratory infections.

## Conclusions

A comparative experimental in vivo study was performed to compare co-infection with *C. parvum* and BCoV to infection with single infections by these pathogens. However, the calves were naturally infected with *C. parvum* on the farm of origin, despite collection of calves within 12 h following birth. This emphasizes how readily and early neonatal calves can become infected with the parasite and the difficulties procuring *C. parvum*-free calves for experimental studies.

Although the samples size in our study was small, the findings indicate that natural followed by experimental infection with *C. parvum* is more debilitating to neonatal calf health than a co-infection with *C. parvum* and BCoV. These findings suggest that there are disparities in clinical outcomes between different co-infections. More information about the interactions between commonly occurring intestinal pathogens of calves would help unravel the complexity of NCD in the cattle industry and thereby provide some evidence-based recommendations on appropriate interventions.

## Supplementary Information

**Additional file 1. Calf breed and sex per group.** Overview of the calves’ breed and sex in the different groups.

**Additional file 2.** **Clinical score table**. Determination of clinical score in calves inoculated with C. parvum, BCoV, or both.Calves with an overall clinical score >3 were classified as sick, and calves with a fecal score >3 were classified as diarrheic.Watery diarrhea (score 4) was classified as severe diarrhea

## Data Availability

The datasets generated during the current study are available from the corresponding author on reasonable request.
