# Supplementary material for: Cryptosporidium parvum and bovine coronavirus in naturally and experimentally exposed calves: clinical outcome and pathogen shedding
Source: Vet Res. 2026 Apr 3;57:63. doi: 10.1186/s13567-026-01725-x (PMC13154558; doi:10.1186/s13567-026-01725-x)
Supplement: Supplementary file 1 — Additional file 1. Calf breed and sex per group. Overview of the calves’ breed and sex in the different groups. [file 13567_2026_1725_MOESM1_ESM.docx]

# Supplementary tables

#### Additional file 1. Calf breed and sex per group

|  | **Holstein-Friesian** | **Holstein-Aberdeen Angus mix** | **Holstein-British Blue mix** |
| --- | --- | --- | --- |
| **Group 1** |  | 1 bull calf | 2 bull calves  2 heifers |
| **Group 2** | 1 bull calf | 1 heifer | 2 bull calves  1 heifer |
| **Group 3** | 3 bull calves |  | 1 bull calf  1 heifer |

**Additional file 1.** Overview of the calves’ breed and sex in the different groups.

#### Additional file 2. Clinical score table.

|  | **0** | **1** | **2** | **3** | **4** |
| --- | --- | --- | --- | --- | --- |
| **Behaviour** | Bright, alert, responsive.  On their feet/rise when entering. |  | Depressed/  Less responsive/ Slow to rise | Markedly depressed/ Unresponsive/  Needs help to rise | Unresponsive to any stimulation/  Falls when helped up |
| **Temperature (°C)** | <39.5 | 39.6 – 39.9 | 40 – 40.4 | >40.5 |  |
| **Appetite** | Strong suckle reflex/  Drinks full meal of milk | Strong suckle reflex/ Interrupted feeding/ Drinks more than ¾ of meal offered | Weak suckle reflex/  Helped feeding/  Drinks more than ½ of meal offered | Chewing movements/ Helped feeding/  Drinks more than ¼ of meal offered | No suckle reflex/  Tube-feeding/  No interest in food |
| **Fecal consistency** | Normal | Pasty |  | Runny (watery with solids in it) | Watery |
| **Hydration status** | Normal skin turgor and normal eyeball position |  | Skin turgor 1-2 sec | Skin turgor 2-5 sec/sunken eyeballs |  |
| **Abdominal pain** | No signs of pain |  | Tense abdomen during palpation |  | Rectal tenesmus |

**Additional file 2.** Determination of clinical score in calves inoculated with *C. parvum*, BCoV, or both. Calves with an overall clinical score >3 were classified as sick, and calves with a fecal score >3 were classified as diarrheic. Watery diarrhea (score 4) was classified as severe diarrhea.

#### Additional file 3. Primers, probes and cycling conditions for PCR-assays.

|  | **Primer/probe** | **Sequence (5’-3’)** | **Cycling conditions** | **Reference** |
| --- | --- | --- | --- | --- |
| ***Cryptosporidium* qPCR assay** | | | | |
| Forward | Cryp18S_Frt | AGTGACAAGAAATAACAATACAGG | 95°C for 10 min.  45 cycles of 95°C for 15s, 60°C for 60s, and 72°C for 30s. | [50] |
| Reverse | Cryp18S_Rrt | CCTGCTTTAAGCACTCTAATTTTC |  |  |
| Probe |  | FAM/ACCAGACTT/ZEN/GCCCTCC/3IABkFQ |  |  |
|  | | | | |
| **Bovine coronavirus RT-qPCR assay** | | | | |
| Forward | BCOV1F20 | TGGTGTCTATATTCATTTCTGCTG | 50°C for 5 min, 95°C for 20s.  40 cycles of 95°C for 15s and 60°C for 60s. | [51] |
| Reverse | BCOV1R89 | GGCCACTGCCTAGGATACA |  |  |
| Probe | BCOV1P48 | FAM/ACACGTCCCTGGCTGAAAGCTG/BHQ1 |  |  |
|  | | | | |
| **Nested PCR assay for *C. parvum* sequencing** | | | | |
| **First PCR** | | | 95°C for 3 min.  45 cycles of 94°C for 45s, 50°C for 45s, and 72°C for 60s.  72°C for 10 min. | [23, 24] |
| Forward | AL3531 | ATAGTCTCCGCTGTATTC |  |  |
| Reverse | AL3535 | GGAAGGAACGATGTATCT |  |  |
| **Second PCR** | | |  |  |
| Forward | AL3532 | TCCGCTGTATTCTCAGCC |  |  |
| Reverse | AL3532 | GCAGAGGAACCAGCATC |  |  |
|  | | | | |
| **PCR assay for *Cryptosporidium* species determination** | | | | |
| Forward | SSU_F3 | GGAAGGGTTGTATTTATTAGATAAAG | 95°C for 3 min.  45 cycles of 94°C for 45s, 55°C for 45s, and 72°C for 60s.  72°C for 10 min. | [25] |
| Reverse | SSU_R3 | AAGGAGTAAGGAACAACCTCCA |  |  |

**Additional file 3.** FAM: 6-carboxyfluorescein. ZEN: internal ZEN™ quencher. 3IABkFQ: Iowa Black ® RQ. BHQ: Black hole quencher.

#### Additional file 4. Sum of days that individual calves presented clinical signs.

| **Group** | **Calf** | **Peak rt (°C)** | **Number of days with** | | | | | | **Peak clinical score** | **Days with clinical score >3** |
| --- | --- | --- | --- | --- | --- | --- | --- | --- | --- | --- |
|  |  |  | Increased rt | Depression | Diarrhea | Decreased appetite | Dehydration | Pain |  |  |
| 1 | 1A | 39.6 | 1 (27) | 0 (27) | 7 (24) | 0 (27) | 1 (26) | 0 (26) | 4 | 1 |
| 1 | 1B* | 39.6 | 1 (21) | 1 (21) | 4 (24) | 0 (21) | 1 (21) | 1 (26) | 6 | 4 |
| 1 | 1C* | 39.7 | 1 (21) | 0 (21) | 4 (24) | 0 (21) | 0 (21) | 0 (26) | 4 | 2 |
| 1 | 1D | 39.6 | 2 (27) | 0 (27) | 3 (25) | 0 (27) | 0 (26) | 1 (26) | 5 | 3 |
| 1 | 1E** | 39.5 | 0 (23) | 0 (23) | 3 (20) | 1 (23) | 0 (21) | 0 (21) | 8 | 2 |
| 2 | 2A | 39.8 | 4 (27) | 1 (27) | 8 (25) | 0 (27) | 0 (26) | 0 (26) | 7 | 3 |
| 2 | 2B | 39.7 | 1 (27) | 0 (27) | 3 (25) | 0 (27) | 2 (26) | 0 (26) | 7 | 1 |
| 2 | 2C | 39.7 | 4 (27) | 5 (27) | 6 (25) | 5 (27) | 5 (27) | 1 (27) | 12 | 6 |
| 2 | 2D | 39.5 | 0 (27) | 0 (27) | 6 (23) | 0 (27) | 0 (26) | 2 (26) | 6 | 2 |
| 2 | 2E | 40.2 | 4 (27) | 1 (27) | 4 (24) | 1 (27) | 4 (26) | 2 (26) | 9 | 5 |
| 3 | 3A | 39.5 | 0 (27) | 0 (27) | 5 (21) | 0 (27) | 1 (27) | 0 (27) | 3 | 0 |
| 3 | 3B | 39.6 | 1 (27) | 0 (27) | 4 (26) | 0 (27) | 0 (27) | 0 (27) | 3 | 0 |
| 3 | 3C | 39.6 | 1 (27) | 0 (27) | 4 (23) | 0 (27) | 1 (27) | 1 (27) | 7 | 3 |
| 3 | 3D | 39.5 | 0 (26) | 0 (25) | 2 (24) | 0 (27) | 0 (26) | 0 (26) | 3 | 0 |
| 3 | 3E | 39.8 | 2 (26) | 0 (25) | 0 (23) | 0 (27) | 2 (26) | 1 (26) | 3 | 0 |

**Additional file 4.** Sum of days individual calves presented clinical signs. Number in brackets: number of total observations per animal for each clinical sign. Rt = rectal temperature. *: Calf 1B, and 1C developed an eye infection, see Section 3.1. **: Calf 1E developed an ear tag infection, see Section 3.1.

Group 1: Natural and experimental *C. parvum* infection*.*

Group 2: Natural and experimental with *C. parvum* and experimental infection with BCoV.

Group 3: Natural infection with *C. parvum* and experimental infection with BCoV.
