# Supplementary figures and images for: Cryptosporidium parvum and bovine coronavirus in naturally and experimentally exposed calves: clinical outcome and pathogen shedding
Source: Vet Res. 2026 Apr 3;57:63. doi: 10.1186/s13567-026-01725-x (PMC13154558; doi:10.1186/s13567-026-01725-x)

## Percentage weight gain in calves from week to week

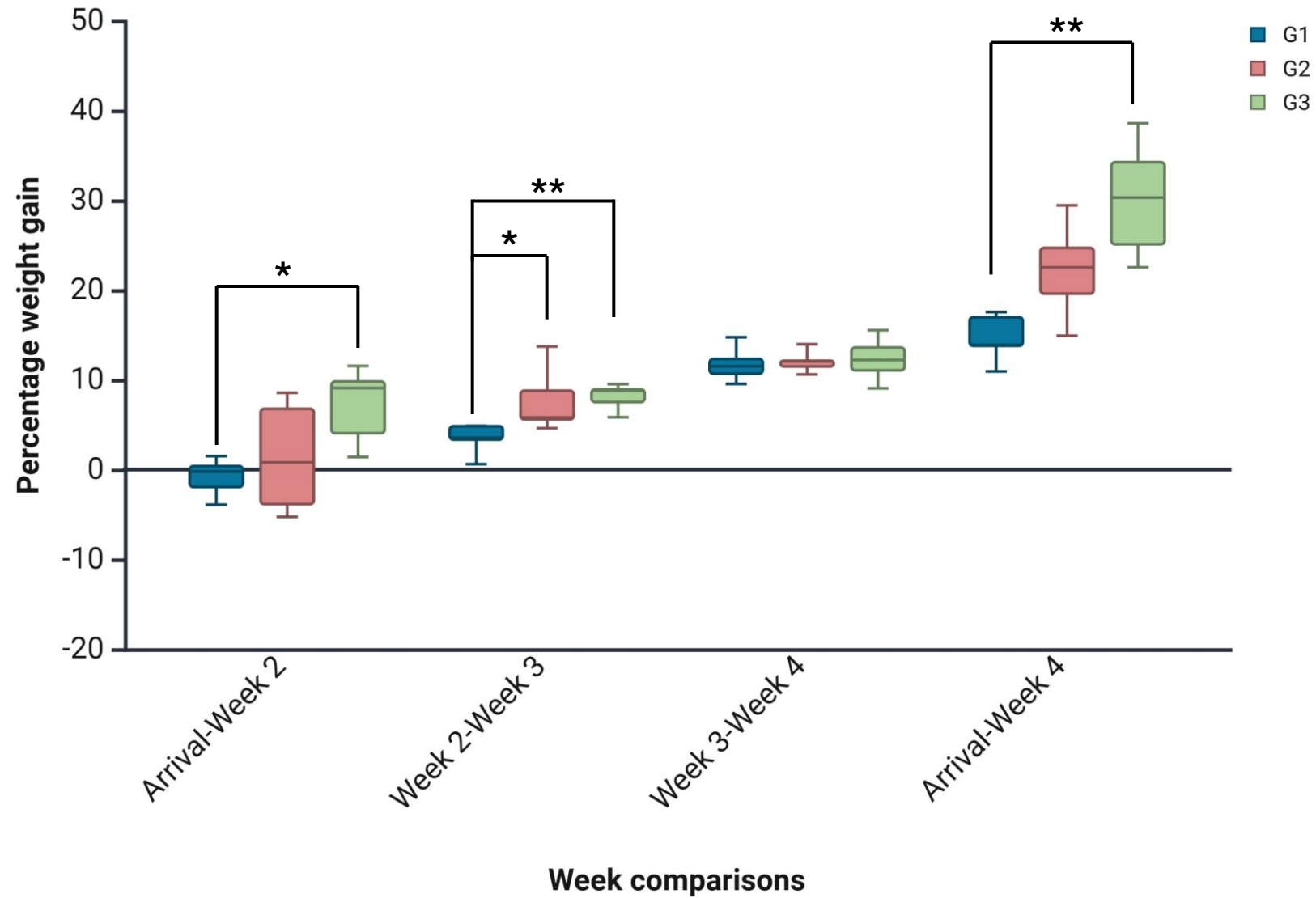

Supplement: Supplementary file 2 — Additional file 2. Clinical score table. Determination of clinical score in calves inoculated with C. parvum, BCoV, or both.Calves with an overall clinical score >3 were classified as sick, and calves with a fecal score >3 were classified as diarrheic.Watery diarrhea (score 4) was classified as severe diarrhea [file 13567_2026_1725_MOESM2_ESM.pdf]
